# The DICER1 familial cancer syndrome: clinical and molecular update

**DOI:** 10.1186/1687-9856-2013-S1-O5

**Published:** 2013-10-03

**Authors:** Cheri Deal

**Affiliations:** 1 Endocrine Service, Centre Hospitalier Universitaire-Ste-Justine/University of Montreal, Quebec, Canada

## 

*DICER1* is an RNase endonuclease important for production of microRNAs, which regulate multiple protein-coding genes involved in growth and development. It has been linked to several tumors including pleuropulmonary blastoma (PPB), cystic nephroma, ovarian Sertoli-Leydig cell tumors and thyroid neoplasia. Most of the manifestations of *DICER1* mutations occur in young children, adolescents and young adults; only thyroid neoplasia are diagnosed throughout adulthood. Although mutations in this gene behave as autosomal dominant tumor suppressor genes, the penetrance of the various tumors is highly variable, rendering screening and genetic counseling challenging.

We have recently hypothesized that mutations in this gene are also responsible for the very rare but often fatal cases of infant (< 3y) Cushing Disease, which can be missed initially because of absent or only minimal signs of intracranial hypertension and hypercortisolemia in this age group. Eye signs, such as strabismus or proptosis, may be the first manifestations of these tumors. The ACTH-producing pituitary tumors seen in these infants have a histopathology and electron microscopy that has been termed pituitary blastoma. The incomplete penetrance of this and the other manifestations of the syndrome makes the involvement of additional predisposing factors highly likely, candidates of which are under investigation.

This presentation will highlight the cardinal features of this new familial cancer syndrome, review the molecular biology of DICER1, consider additional polygenic influences on tumor development and discuss the current recommendations for following these families.

